# Expression of c-Myc, Bcl2, Bcl6, and Cyclin D1 in High-Grade B-Cell Lymphoma

**DOI:** 10.7759/cureus.29527

**Published:** 2022-09-24

**Authors:** Seemal Ali, Zonaira Rathore, Fizza Jahangir, Zubaria Rafique, Akhtar S Chughtai, Aribah Atiq

**Affiliations:** 1 Histopathology, Chughtai Institute of Pathology, Lahore, PAK

**Keywords:** double expressor, triple expressor, hgbl, high grade b cell lymphoma, c-myc, cyclin d1

## Abstract

Background

High-grade B-cell lymphomas (HGBLs), with c-Myc, Bcl2, and/or Bcl6 rearrangement, are aggressive neoplasms with poor clinical outcomes. Cyclin D1 is a proto-oncogene that is generally expressed by mantle cell lymphoma, its variants, and many other neoplasms.

Aim

The aim of this study is to investigate the expression and frequency of cyclin D1 in HGBL and its association with double expressor and triple expressor lymphomas. A few pieces of research have been reported on the expression of cyclin D1 in triple-hit lymphoma, renaming it quadruple-hit lymphoma. In the present study, we also used immunohistochemical (IHC) stains to look at the expression of cyclin D1 in double expressor and triple expressor.

Methodology

This is a cross-sectional descriptive study involving 60 cases, including both double and triple expressors, diagnosed by using hematoxylin and eosin (H&E) staining with subsequent IHC stains. We analyzed the expression of cyclin D1 in double expressor and triple expressor.

Results

The expression and frequency of cyclin D1 were interpreted in terms of positivity in double expressors and triple expressors. Cyclin D1 positivity was observed in three (5%) cases of double expressors and two (3.33%) cases of triple expressors. Overall, five (9%) cases of HGBL were positive for cyclin D1.

Conclusion

This study showed a very low frequency of cyclin D1 expression in double and triple expressor lymphomas. However, a few cases of HGBL showed expression of cyclin D1 and were not limited to only mantle cell lymphoma and its variants.

## Introduction

High-grade B-cell lymphomas (HGBLs) encompass a variety of diseases with different immunophenotypic variants as well as molecular subtypes. The World Health Organization (WHO) updated its categorization of lymphoid neoplasms in 2016 to reflect significant developments in lymphoma biology since 2008 [1]. The WHO classification of lymphoid neoplasms further categorizes HGBL with the addition of a new term, "high-grade B-cell lymphoma with c-Myc, Bcl2, and/or Bcl6 rearrangement" [2]. The term "double expressor" refers to HGBL that shows c-Myc and Bcl2/Bcl6 immunohistochemical (IHC) expression, whereas the term "triple expressor" refers to HGBL that shows additional Bcl6 expression along with c-Myc and Bcl2. These lymphoid neoplasms are aggressive, with poor clinical outcomes [3]. Now, a few studies are being conducted at an international level, showing the expression of cyclin D1 in triple expressors, regarding them as "quadruple-hit lymphoma" [4]. These neoplasms are more aggressive, with dismal outcomes. Initially, prognostic factors depend upon the clinical stage and proliferative index of lymphoid neoplasm, but with the advancement in molecular studies, clinical outcomes, and new treatment strategies, the requirement for further categorization is now more important than ever.

Among non-Hodgkin’s lymphomas (NHL), HGBL is the most common type, accounting for 30-40% [5]. In Pakistan, exact statistics regarding the incidence of HGBL are unknown, as there is no cancer registry at the national level. According to one study conducted in Pakistan [6], the frequency of double-expressers is 14%. However, no data are available regarding the frequency of triple expressors in our population. Cyclin D1 expression is known to be expressed by mantle cell lymphoma (MCL), particularly pleomorphic and blastoid variants [6]. Recently, Cyclin D1 expression has been seen in a few cases of HGBL, but no data are available regarding its expression in our population [7]. The purpose of this study is (1) to look at the expression of Cyclin D1 in HGBL, (2) the frequency of cyclin D1 in a selected number of cases, and (3) to identify the co-expression of proteins involved in cyclin D1 with c-Myc, Bcl2, and Bcl6 in HGBL.

## Materials and methods

This study was carried out at the Chughtai Institute of Pathology in Lahore, Pakistan, after taking approval from Chughtai Institute of Pathology Institutional Review Board (Reference letter no CIP/IRB/1074) and is a cross-sectional, descriptive study. Cases were extracted from the archives of Chughtai Institute of Pathology from the histopathology department through Nexus Software (Kirtland, OH). Over the course of two years, from January 2019 to January 2021, a cohort of 60 cases was selected by a convenient sampling technique and blindly reviewed by two pathologists with a special interest in hematopathology. All the cases selected in this study were diagnosed as HGBL on the basis of morphology as high-grade non-Hodgkin lymphoma with immunoblastic morphology and subsequent IHC panel. Specimen selection includes mostly excisional biopsies and Tru-Cut biopsies diagnosed as HGBL with double/triple expressers based on morphology and IHC. All the cases of low-grade lymphoma, Burkitt’s lymphoma, transformed follicular lymphoma, and non-contributory IHC results have been excluded from the study.

**Figure 1:**
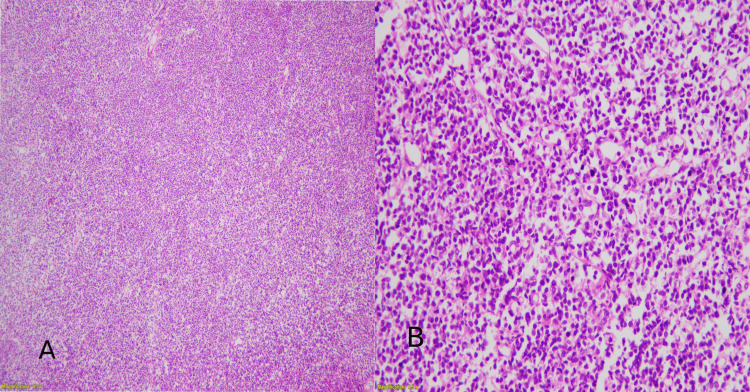
(A) H&E image shows HGBL at 40x magnification. (B) H&E image shows HGBL at 100x magnification. H&E: hematoxylin and eosin; HGBL: high-grade B-cell lymphoma

All tissue samples received were fixed in 10% neutral, buffered formalin in the department of Histopathology, then processed for hematoxylin and eosin (H&E) staining and subsequent IHC markers. Sections were cut at 4-5 µm thickness and stained with H&E (Figure 1).

**Figure 2:**
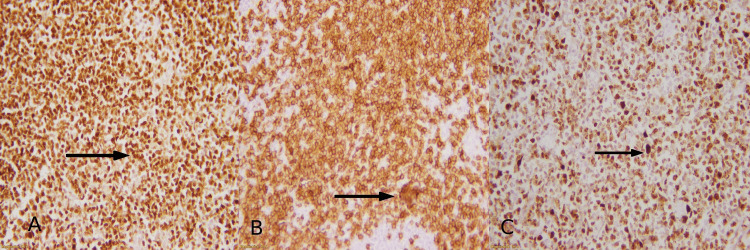
IHC profile of HGBL Diffuse membranous staining of CD20 in tumor cells at (A) 40x magnification and (B) 100x magnification, (C) Nuclear staining of ki-67 >80% IHC: immunohistochemical; HGBL: high-grade B-cell lymphoma

For the application of IHC markers, dewaxing in xylene and rehydration in ethanol are performed. Antigen retrieval in a citrate buffer at 100 °C for 30 minutes is performed with subsequent incubation in 5% bovine serum albumin (BSA) at room temperature for 30 minutes. IHC markers were applied following a standard immunostaining protocol using automated tissue (Peloris, Leica, Germany). Primary antibodies from DAKO (Glostrup, Denmark), CD20 (Clone L26, monoclonal, mouse anti-human), CD3 (polyclonal, rabbit anti-human), and Ki67 (MIB-1, monoclonal, mouse anti-human), were used to classify it as a high-grade, B-cell NHL (Figure 2).

**Figure 3:**
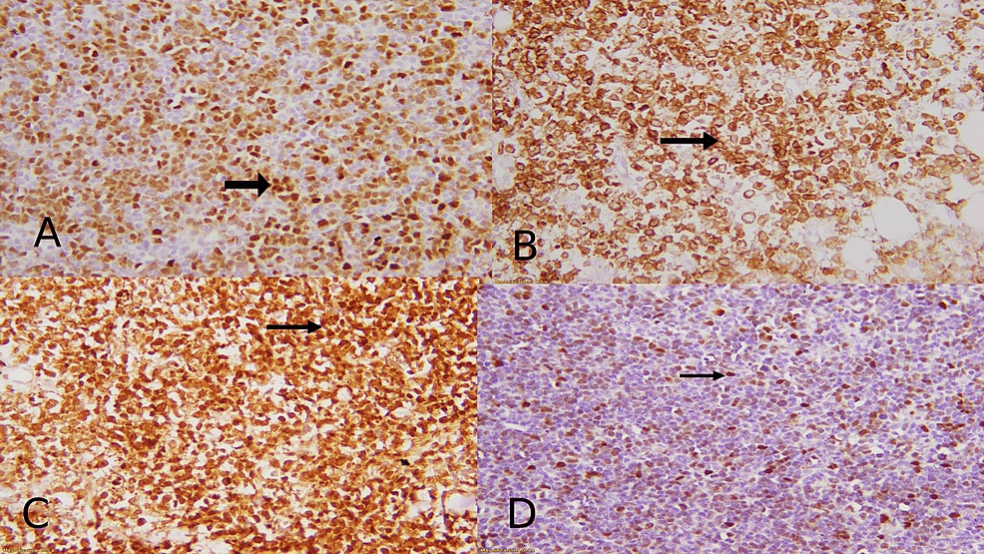
IHC profile shows (A) nuclear staining of c-Myc in 40% of tumor cells; (B) membranous staining of Bcl2 in 20% of tumor cells; (C) nuclear staining of Bcl2 in 10% of tumor cells; (D) nuclear staining of cyclin D1 in tumor cells (10%). IHC: immunohistochemical

Myc (Clone EP121, Rabit Monoclonal primary antibody) from Cell Marque, California, USA. Bcl2 (Clone 124, Monoclonal, Mouse anti-human), Bcl6 (Clone PG-B6p, Monoclonal, Mouse, anti-human), and cyclin D1 (Clone EP12, Monoclonal, anti-human), CD23 (Clone DAK-CD23, Monoclonal, Mouse, anti-human), CD5 (Clone 4C7, Monoclonal, Mouse, anti-human, CD10 (Clone 56C6, Monoclonal, Mouse, anti-human) and MUM1 (Clone MUM1, Monoclonal, Mouse, anti-human) from DAKO, Glostrup, Denmark are also used. The positivity of immunostaining was detected by the percentage of positive cells. Tumor cells were positive for CD20, Bcl2, Bcl6, and cyclin D1 and negative for CD5, CD23, CD10, and MUM1. Ki 67 showed a proliferative index upto 80%. For c-Myc staining, 40% of tumor cells showed nuclear reactivity; 20% of membranous staining by tumor cells for Bcl2 and 10% of nuclear staining for Bcl6 are considered positive. The criteria set for cyclin D1 positivity required nuclear staining in at least 10% of tumor cells. Sections were mounted and examined under a microscope and their expression was then interpreted as positive or negative (Figure 3).

Molecular studies were not performed, as we lack this facility in our setup. Statistical Package of Social Sciences (SPSS) software version 22 (IBM Corp., Armonk, NY) was used to examine the results and conduct statistical analysis. Where necessary, the chi-square test was used. The p-value was calculated and considered significant if ≤ 0.05.

## Results

In this study, out of a total of 60 cases of HGBL, 33 (55%) were males and 24 (45%) were females. The age range was 36-80 years, with a mean age of 53.2 11.3. Of these cases, 28 cases of HGBL were from the cervical lymph node (46.8%), followed by eight cases of axillary lymph nodes (13.4%), seven inguinal (11.7%), four supraclavicular (6.7%), three abdominal (5%), two tonsillar (3.3%), two posterior cervical (3.3%), and two from the posterior pharyngeal (3.3%) lymph nodes. Out of these 60 cases, 48 (80%) were double expressers and showed positive staining for either Bcl2 or Bcl6. Twenty-two (36.7%) cases showed only Bcl2 positivity along with c-Myc while 26 (43.3%) cases were negative for Bcl2. Bcl6 positivity was seen in 27 (45%) cases while 21 (35%) cases were negative for Bcl6 (Table 1).

**Table 1:** Expression of cyclin D1 in double-expressor lymphoma IHC: immunohistochemical

Expression of IHC markers	Positive	Negative	Total
Bcl2 n (%)	22 (36.7%)	26 (43.3%)	48 (80%)
Bcl6 n (%)	27 (45%)	21 (35%)	48 (80%)
cyclin D1 n (%)	3 (5%)	57 (95%)	60 (100%)

Twelve (20%) cases were triple expressors, revealing positive staining for both Bcl2 and Bcl6 (Table 2). All 60 (100%) cases were c-Myc positive.

**Table 2:** Expression of cyclin D1 in triple-expressor lymphoma IHC: immunohistochemical

Expression of IHC markers	Positive	Negative	Total
Bcl2-Bcl6 n (%)	12 (20%)	0	12 (20%)
cyclin D1 n (%)	2 (3.33%)	58 (96.6%)	60 (100%)

Expression of cyclin D1 was seen in only five (9%) cases, including both double expressors and triple expressors. Fifty-five (91.6%) cases showed negative expression for cyclin D1 (Table 3).

**Table 3:** Expression of cyclin D1 in double and triple-expressor lymphoma *Chi-square test was applied; p-value = not statistically significant

Expressors	Cyclin D1 expression n (%)	p-value*
Double expressor n(%)	2(3.33%)	.259
Triple expressor n(%)	3 (5%)	
Total	5 (9%)	

Additionally, all five (9%) cases positive for cyclin D1 were further evaluated in terms of strength as weak, moderate, and strong, and then we compared the strength with double expressors and triple expressors (Table 4), and it showed variable expression.

**Table 4:** Intensity of cyclin D1 in double and triple expressor lymphoma

Cases	Cyclin D1 intensity	Bcl2	Bcl6	Expressor
Case1	Weak	Positive	Negative	Double
Case2	Strong	Negative	Positive	Double
Case3	Weak	Positive	Positive	Triple
Case4	Moderate	Positive	Negative	Double
Case5	Strong	Positive	Positive	Triple

## Discussion

Diffuse large B-cell lymphoma (DLBCL) and Burkitt lymphoma (BL) are clinically and physiologically separate from high-grade B-cell lymphoma (HGBL), a recently discovered category [8]. Patients with HGBL typically exhibit advanced disease, extranodal disease, involvement of the bone marrow, an elevated lactate dehydrogenase level, and a high international prognostic score [9]. According to the recent WHO classification of lymphoid neoplasms (2016), HGBLs with c-Myc, Bcl2, and/or Bcl6 rearrangement were classified as double expressors and/or triple expressors, respectively [10]. Cyclin D1 expression is characteristic of MCL [11]. Cyclin D1 expression can also be seen in other lymphoid malignancies, for example, pleomorphic and blastoid variants of MCL, plasma cell myelomas, and cases of chronic lymphocytic leukemia. Cyclin D1 promotes cell proliferation and cell cycle progression [12]. Cyclin D1, which has a role in stimulating the G1/S transition of the cell cycle, is typically overexpressed in mantle cell lymphoma and has been regarded as a crucial oncogene in this disease [13]. Cyclin D1 positivity indicates that there are some genetic alterations at the molecular level and genetic testing should be done to evaluate them. There are several methods to detect these alterations using IHC markers, flow cytometry, and PCR.

In the present study, we evaluated the expression of cyclin D1 at the protein level by IHC markers and evaluated its association with double expressor and triple expressor. Many studies in the form of case series and case studies showed the expression of cyclin D1 in HBGL. The reported frequency is 1.5-4.3% in previously reported studies [14-16]. However, to date, very limited data are available at the international level regarding cyclin D1 expression in triple-hit lymphoma to label it as quadruple-hit lymphoma [6]. In this study, the frequency of cyclin D1 expression was studied, and it was found that in double expressors, the frequency of cyclin D1 is 5%. While in triple expressors, the frequency is 3.33%, and hence, we can label them as quadruple expressors rather than quadruple-hit lymphoma, as only IHC studies were performed. Cyclin D1 expression is not associated with only triple expressors, but it can also show positivity in double expressors. We calculated a p-value that is insignificant (p = 0.259), and hence, there is no association of double expressor or triple expressor with cyclin D1 expression. Overall, the expression of cyclin D1 is 9% in HGBL. Our study is also in line with the study by Mats et al., which only found cyclin D1 expression in HGBL (particularly DLBCL), as this study proved the fact that cyclin D1 positivity can be seen in HGBL [17]. However, our results are slightly discordant with another study conducted by Vela-Chávez et al., which showed a positivity rate of cyclin D1 in HGBL is 15% [11]. These results were higher than our study. Our study results are concordant with another study by Ittel et al., which found only two cases showing the positive expression of cyclin D1 in triple-hit lymphomas. In our study, only two cases of triple expressor were positive for cyclin D1. We further evaluated the intensity of cyclin D1 antibody derived from DAKO which showed variable results. This finding was discordant with the study of Mats et al., who found weak expression of cyclin D1 derived from the same company as used in this study [17].

This study has some limitations that restrict further evaluation. Due to a lack of molecular research in our setup, we did not conduct fluorescence in situ hybridization (FISH) investigations or mRNA analysis like many other reported studies. Additionally, because it was a single-center study, the results might not accurately reflect the frequency of cyclin D1 expression in high-grade B-cell lymphoma.

## Conclusions

HGBL is a set of heterogeneous disorders with numerous genetic mutations, poor care outcomes, and demanding therapy. Since cyclin D1 expression is frequently observed in MCL (especially the pleomorphic and blastoid types), pathologists should always take HGBL, particularly DLBCL, into account when making a diagnosis of MCL. As cyclin D1 is linked to a poor prognosis for patients, its expression may result in a misleading lymphoma diagnosis in cases of high-grade morphology. However, given that there is no conclusive correlation between double and triple expressors, we do not advise reflex cyclin D1 testing for them. But because quadruple hit lymphomas have a bad prognosis, instances with the expression of this marker should be investigated for the possibility of this entity. To prevent this diagnostic confusion, the patient needs to undergo even more testing.
